# Thermomechanical Characterization and Modeling of NiTi Shape Memory Alloy Coil Spring

**DOI:** 10.3390/ma16103673

**Published:** 2023-05-11

**Authors:** Jesús G. Puente-Córdova, Flor Y. Rentería-Baltiérrez, José M. Diabb-Zavala, Nasser Mohamed-Noriega, Mario A. Bello-Gómez, Juan F. Luna-Martínez

**Affiliations:** 1 Facultad de Ingeniería Mecánica y Eléctrica, Universidad Autónoma de Nuevo León, Av. Universidad s/n Cd. Universitaria, San Nicolás de los Garza 66455, Mexico; 2 Facultad de Ciencias Químicas, Universidad Autónoma de Nuevo León, Av. Universidad s/n Cd. Universitaria, San Nicolás de los Garza 66455, Mexico

**Keywords:** shape memory, nickel-titanium, thermomechanical, fractional calculus, Fractional Zener Model, damping

## Abstract

Today, shape memory alloys (SMAs) have important applications in several fields of science and engineering. This work reports the thermomechanical behavior of NiTi SMA coil springs. The thermomechanical characterization is approached starting from mechanical loading–unloading tests under different electric current intensities, from 0 to 2.5 A. In addition, the material is studied using dynamic mechanical analysis (DMA), which is used to evaluate the complex elastic modulus E* = E^′^ − iE^″^, obtaining a viscoelastic response under isochronal conditions. This work further evaluates the damping capacity of NiTi SMA using tan δ, showing a maximum around 70 °C. These results are interpreted under the framework of fractional calculus, using the Fractional Zener Model (FZM). The fractional orders, between 0 and 1, reflect the atomic mobility of the NiTi SMA in the martensite (low-temperature) and austenite (high-temperature) phases. The present work compares the results obtained from using the FZM with a proposed phenomenological model, which requires few parameters for the description of the temperature-dependent storage modulus E^′^.

## 1. Introduction

Nowadays, shape memory alloys (SMAs), commonly known as smart materials, are widely used in diverse applications, such as mechanical, aerospace, automotive, and biomedical devices [1,2,3,4,5]. These materials, after being exposed to a load that plastically deforms them beyond their elastic limit or yield strength, can return to their original state when heated. In addition, these alloys are also recognized for their superelasticity and high damping capacity. While most metals show plastic deformation from 0.2% elongation, SMAs can show 6% elongation without reaching the plastic zone. The shape memory effect and superelasticity present in these alloys are due to a reversible phase transformation in the solid state from B19′ martensite to B2-austenite, with or without the R-phase [1,6]. In other words, the sample deforms during cooling and recovers during heating; these characteristics are due to the phase change called thermoelastic martensitic transformation. In addition, it is important to mention that the combined mechanical properties obtained in SMAs depend on the chemical composition and the structure formed during thermomechanical processing [7,8,9]. Several alloys with shape memory and superelasticity are reported in the literature, but only a few have been commercially developed, such as NiTi, NiTiX (where X is a ternary element), and CuZnAl [10,11]. New applications are based on NiTi, NiTiCu, and NiTiNb; however, other alloys, for example CuAlNi or FeMnSi, are starting to break into the market, while others such as NiAl or NiTiZr are of potential interest, despite being brittle. Alloys with Pt as the base element attract attention because NiTi and CuZnAl alloys can only be used up to around 100 °C. Therefore, alloys that can operate at higher temperatures are required [12,13].

Several engineering applications with an SMA require a specific geometric design. Moreover, this geometry needs a process called training, with the purpose of optimizing the shape memory effect in a specific spatial direction. A practical method that is followed in the literature consists of heat treating the SMA above its final austenitic transformation temperature. Under these thermal conditions, the SMA is maintained at the desired shape during a fixed interval of time; the SMA is then cooled rapidly to obtain a twinned martensite phase [14,15].

A geometry widely used in commercial and practical applications is the coil spring, which has good performance as an actuator for designing mechanical and mechatronic systems. In this regard, Aguiar et al. [16] undertook an experimental analysis of vibration reduction using SMA coil springs. SMA elements introduce complex behaviors to the dynamic system. They reported that adaptability due to temperature variations is defined by a competition between changes in stiffness and hysteretic behavior. Cho et al. [17] studied an anti-freezing system using NiTiCo SMA coil springs that operates near sub-zero temperatures to prevent freezing accidents in water pipes. Holanda et al. [15] studied the complex stiffness k* of a mechanical system having 1 degree-of-freedom with a NiTi SMA coil spring actuator. The dynamic response of the system was evaluated, with potential application in controlling mechanical vibrations. De melo-Santiago et al. [18] studied the thermomechanical behavior of superelastic NiTi SMA helical extension springs manufactured by investment casting. The mechanical behavior revealed that the maximum force developed in the spring increases linearly as a function of temperature. Shimoga and coworkers [6] presented a technical note, where the characteristics and properties of NiTi helical springs are highlighted, suggesting uses in biomedical, robotics, and advanced high-tech applications.

Motivated by these findings, the aim of the present work focuses on studying the thermomechanical behavior and modeling of tensile NiTi SMA coil springs, with potential applications as actuators in mechanical systems. Then, reverse transformation of NiTi SMA wire was analyzed by dynamic mechanical analysis (DMA). For a better understanding of the viscoelastic results, the complex elastic modulus E* was analyzed under the framework of fractional calculus, using the Fractional Zener Model (FZM).

## 2. Materials and Methods

### 2.1. Experimental Methodology

Commercial NiTi SMA coil springs were used in this study with a coil diameter of 0.75 mm, external diameter of 6.4 mm, 18 coils, and a spring index of 7.533 (ratio between the median diameter and wire diameter); it was manufactured by Dynalloy Inc., Irvine, CA, USA. A commercial steel spring with a coil diameter of 0.80 mm, external diameter of 12.7 mm, 20 coils, and a spring index of 14.875 was used only as a comparison for mechanical tests.

An important aspect in the study of SMAs is knowing the atomic composition. For this, semiquantitative elemental analysis was carried out using energy dispersive X-ray spectroscopy (EDX). Table 1 shows the obtained EDX results, revealing the presence of Ni and Ti with a near-equiatomic composition.

To determine the temperatures of the martensitic transformation, differential scanning calorimetry (DSC) analysis was carried out on a DSC-Q2000 device from TA Instruments. For this, small samples of 8 mg were cut from springs, and they were placed in aluminum pans. Before the measurement, heating was carried out at 200 °C to erase a possible thermomechanical history inherent to the handling of the alloy. Then, the samples were subjected to a thermal cycle (cooling–heating) in the temperature range between 30 °C and 120 °C at a rate of 10 °C/min.

**Figure 1:**
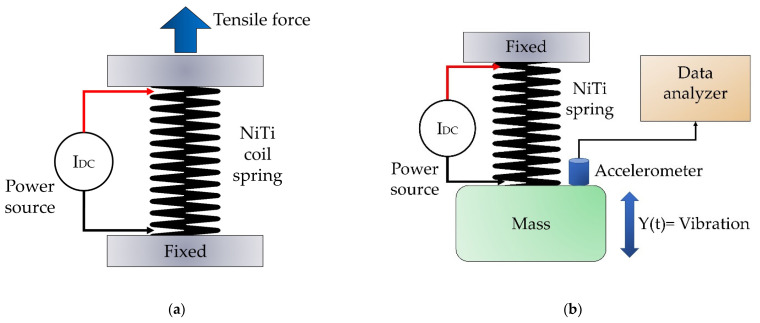
Schematic setup for (**a**) thermomechanical tests and (**b**) vibratory tests.

Thermomechanical characterization was carried out in two stages. In the first stage, the mechanical response of the springs was evaluated through experimental tests using a Shimadzu AGS-X tensile-testing machine with a load cell of 10 kN. The measurements were made at the crosshead speed of 10 mm/min at a temperature of 23 °C; a preload of 1 N was applied to the coil springs before starting the test. For the loading–unloading cycles, the same conditions were used, and a BK Precision 1621A DC power supply was used to generate different temperature states; see Figure 1a. All the tests were replicated at least three times to achieve reproducibility.

In the second stage, a small sample of NiTi SMA was taken in the form of wire with a diameter equal to 0.25 mm and a length of 25 mm (Dynalloy Inc., Irvine, CA, USA) to perform dynamic mechanical analysis (DMA). For this, a 8000 Perkin Elmer DMA device was used. The analysis conditions were a frequency of 0.5 Hz, a temperature range from 20 to 120 °C, and a heating rate of 2 °C/min to obtain a uniform temperature distribution in the material. The studied samples were analyzed in the tension mode and under an amplitude of 5 µm. Results were recorded using the mathematical formalism of the complex elastic modulus E* = E^′^ − iE^″^ and analyzed by a fractional calculus approach, using the FZM.

As a contribution of this work, a 1 degree-of-freedom mass-spring NiTi system in a vertical position was built (Figure 1b). Two states of operation were defined: “A” the system at room temperature (0 A) and “B” the system after being electrically heated with 2.5 A in direct current (DC) and controlled voltage (1.68 V). The test mass used was 261.8 g. Although there are several effects to consider, in this work we were able to use a simple approach that allows us to give an overview of the use of nonlinear springs in the design of vibratory systems. The dynamic behavior was evaluated through the measurement of free vibration using a Brüel & Kjær accelerometer; the data were obtained with a Data Physics Quattro analyzer.

### 2.2. Fractional Calculus and Fractional Zener Model

Calculus of arbitrary order, also known as fractional calculus, is a branch of mathematics that is based on the use of differential and integral operators of arbitrary order, in which the order can be an integer, fraction, or complex number. In recent years there has been an increase in the number of publications that report on the use of this mathematical tool to address various problems related to science and engineering [19,20,21,22,23]. Nowadays, fractional calculus has been applied successfully to the study of complex viscoelastic materials. To obtain adequate fittings of experimental data, analogous mechanical models and generalized models are used. However, these models have the disadvantage of using many parameters. The use of models with fractional order operators (derivatives and integrals) offers an adequate description, with a minimum number of parameters. For this purpose, and to obtain a mechanical response, the springpot is used (also known as the Scott-Blair element). The constitutive equation that describes the springpot is $\sigma ={E\tau }^{\alpha }D_{t}^{\alpha }\gamma$, where σ is the stress, γ is the strain, E is the elastic modulus, and τ is the relaxation time: the ratio between viscosity η and E. The operator $D_{t}^{\alpha }$ represents the fractional derivative (for example Riemann–Liouville, Caputo, or Grünwald–Letnikov), where the fractional order α takes values between 0 and 1. The springpot intimately combines the solid behavior or Hookean spring (α=0), with liquid behavior or Newtonian dashpot (α=1).

**Figure 2:**
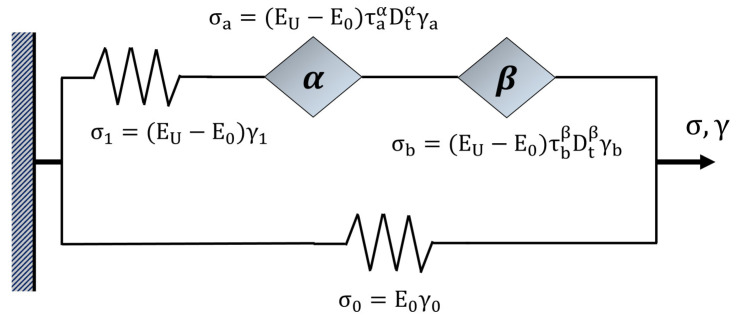
The Fractional Zener Model (FZM).

To obtain a rheological model capable of describing experimental results, the classical Zener model is modified with two springpots to obtain the so-called Fractional Zener Model or FZM (see Figure 2). It has been reported that the fractional order parameters of the FZM are related to the cooperative molecular mobility of the mechanical relaxation phenomena in polymers [19,24,25]. Puente-Córdova et al. [26] reported the use of the FZM to model the viscoelastic behavior of isotropic and anisotropic magnetorheological elastomers. In an innovative way, using the FZM, Reyes-Melo et al. [27] studied the mechanical response of an SMA ribbon during a reverse transformation.

Figure 2 shows the three components that describe the Fractional Zener Model (FZM). First, the springpot, “α”, characterizes short times (τ_a_) associated with viscoelastic behavior in the region at high frequencies or low temperatures. The springpot, “β”, characterizes long times (τ_b_) associated with viscoelastic behavior in the region at low frequencies or high temperatures, and the two spring elements represent the elastic response of the material. E_U_ is the unrelaxed modulus corresponding to the values of E^′^ at high frequencies or low temperatures, while E_0_ is the relaxed modulus corresponding to values of E^′^ at low frequencies or high temperatures.

Therefore, from the constitutive equations of spring and springpot elements exhibited in Figure 2, and considering the global stress σ and the global strain γ, the fractional differential equation for the FZM can be written as:

(1) $$\left(E_{U}-E_{0}\right)\gamma =\left(\sigma -E_{0}\gamma \right){+\tau }_{b}^{-\beta }D_{t}^{-\beta }\left(\sigma -E_{0}\gamma \right){+\tau }_{a}^{-\alpha }D_{t}^{-\alpha }\left(\sigma -E_{0}\gamma \right)$$

In this work the operators $D_{t}^{-\alpha }$ and $D_{t}^{-\beta }$ are described with the Riemann–Liouville definition [28,29], with fractional orders that satisfy 0<α<β<1. From Equation (1) and considering that the NiTi SMA in DMA is under a sinusoidal mechanical stimulus, the elastic complex modulus E* = E^′^ − iE^″^ can be calculated as a function of the angular frequency ω at a constant temperature by applying the Fourier transform [20,26]. Equation (2) corresponds to E* derived from Equation (1). From Equation (2), the Equations (3) and (4) were obtained and correspond to the mathematical expressions for storage modulus E^′^ and loss modulus E^″^, respectively. The loss factor or tangent delta (tan δ) is obtained from the ratio E^″^/E^′^.

(2) $$E^{*}\left(i\omega \right)=E^{\prime }-{\mathrm{iE}}^{\prime \prime }=\frac{E_{U}{+E}_{0}\left[{\left({i\omega \tau }_{a}\right)}^{-\alpha }+{\left({i\omega \tau }_{b}\right)}^{-\beta }\right]}{1+{\left({i\omega \tau }_{a}\right)}^{-\alpha }+\;{\left({i\omega \tau }_{b}\right)}^{-\beta }}$$

(3) $$E^{\prime }=E_{o}+\frac{\left(E_{U}-{\;E}_{0}\right)\left[1+{\left[{\omega \tau }_{a}\right]}^{-\alpha }\cos\left(\alpha \frac{\pi }{2}\right)+{\left[{\omega \tau }_{b}\right]}^{-\beta }\cos\left(\beta \frac{\pi }{2}\right)\right]}{{\left[1+{\left[{\omega \tau }_{a}\right]}^{-\alpha }\cos\left(\alpha \frac{\pi }{2}\right)+{\left[{\omega \tau }_{b}\right]}^{-\beta }\cos\left(\beta \frac{\pi }{2}\right)\right]}^{2}+{\left[{\left[{\omega \tau }_{a}\right]}^{-\alpha }\sin\left(\alpha \frac{\pi }{2}\right)+{\left[{\omega \tau }_{b}\right]}^{-\beta }\sin\left(\beta \frac{\pi }{2}\right)\right]}^{2}}$$

(4) $$E^{\prime \prime }=\frac{\left(E_{0}-{\;E}_{U}\right)\left[{\left[{\omega \tau }_{a}\right]}^{-\alpha }\sin\left(\alpha \frac{\pi }{2}\right)+{\left[{\omega \tau }_{b}\right]}^{-\beta }\sin\left(\beta \frac{\pi }{2}\right)\right]}{{\left[1+{\left[{\omega \tau }_{a}\right]}^{-\alpha }\cos\left(\alpha \frac{\pi }{2}\right)+{\left[{\omega \tau }_{b}\right]}^{-\beta }\cos\left(\beta \frac{\pi }{2}\right)\right]}^{2}+{\left[{\left[{\omega \tau }_{a}\right]}^{-\alpha }\sin\left(\alpha \frac{\pi }{2}\right)+{\left[{\omega \tau }_{b}\right]}^{-\beta }\sin\left(\beta \frac{\pi }{2}\right)\right]}^{2}}$$

To obtain isochronal conditions for the FZM, we consider the relaxation times for cooperative motions, τ_coop_, that verify a power law in a temperature range T_0_ < T < T* [24,25].

(5) $$\tau_{\mathrm{coop}}{=\tau }_{0}{\left[\exp\left(\frac{E_{a}}{k_{B}T}\right)\right]}^{Z}$$

With Z = T(T* − T_0_)/T*(T − T_0_), where E_a_ is the activation energy corresponding to elementary movements of the cooperative mobility; k_B_ is the Boltzmann constant; and T is the absolute temperature. τ_0_ is a pre-exponential factor with values within the range of 10^−16^ s ≤ τ_0_ ≤ 10^−13^ s [19,20]. Then, T* is a crossover temperature above Z = 1. Below T*, the relaxation times of cooperative motions verify the empirical Vogel–Fulcher–Tammann (VFT) equation. T_0_ is a temperature where both Z and τ_coop_ extrapolate to infinite values. It should be noted that the FZM parameters are obtained from the experimental results; see ref. [19,20,26].

## 3. Results and Discussion

### 3.1. Thermomechanical Characterization

**Figure 3:**
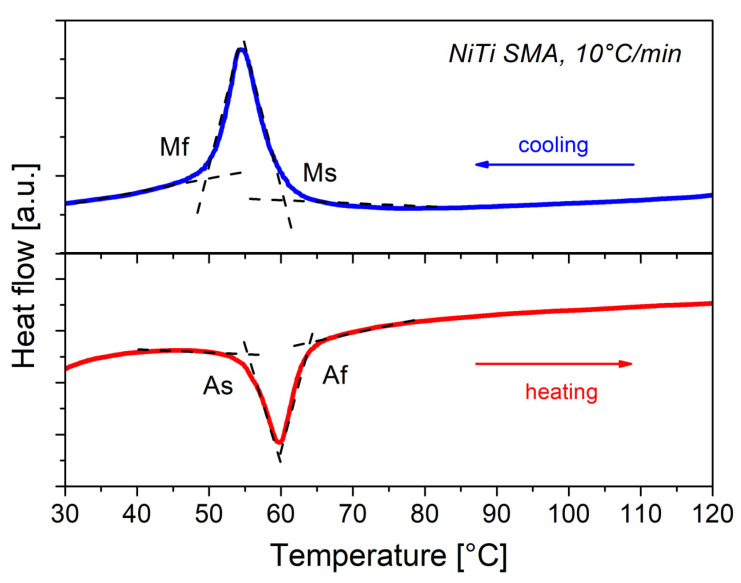
DSC results for the NiTi SMA.

From a practical point of view, it is important to obtain the martensitic transformation temperatures of the SMAs. Figure 3 shows the differential scanning calorimetry (DSC) results, in which the reverse martensitic transformation and the martensitic transformation are observed during the heating–cooling processes. The presence of the R-phase is not detected. The transformation temperatures were calculated: austenite transformation start As = 55.3 °C, austenite transformation finish Af = 64 °C, martensite transformation start Ms = 60.5 °C, and martensite transformation finish Mf = 49.5 °C.

**Figure 4:**
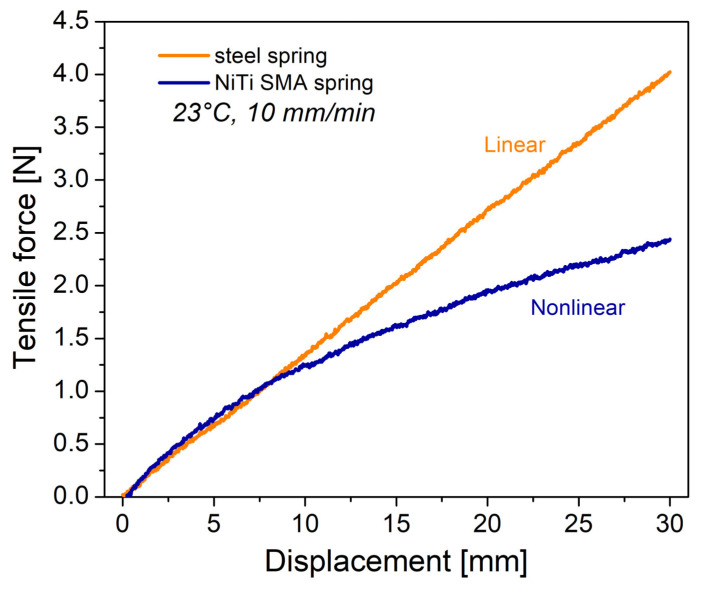
Mechanical response for steel spring and NiTi SMA spring.

The results of tensile tests for the steel spring and the NiTi SMA spring are presented in Figure 4. A linear behavior is clearly exhibited by the steel spring, following Hooke’s law. Conversely, the NiTi SMA spring exhibits a non-linear behavior, due to the twinned martensite phase [15]. When an external force acts on the spring, the macroscopic deformation is produced by the movement of the twin limits at the martensite phase. This means that small displacements at the atomic level, when they occur in a preferential direction, generate an important change in the mechanical response. Based on the results of Figure 4, at low displacement, the stiffness K of the springs was calculated. A value of 0.135 N/mm was obtained for the steel spring, while for the NiTi SMA spring 0.160 N/mm was obtained. However, to entirely describe the mechanical response of the NiTi SMA spring, it is necessary to establish a nonlinear equation or constitutive model.

**Figure 5:**
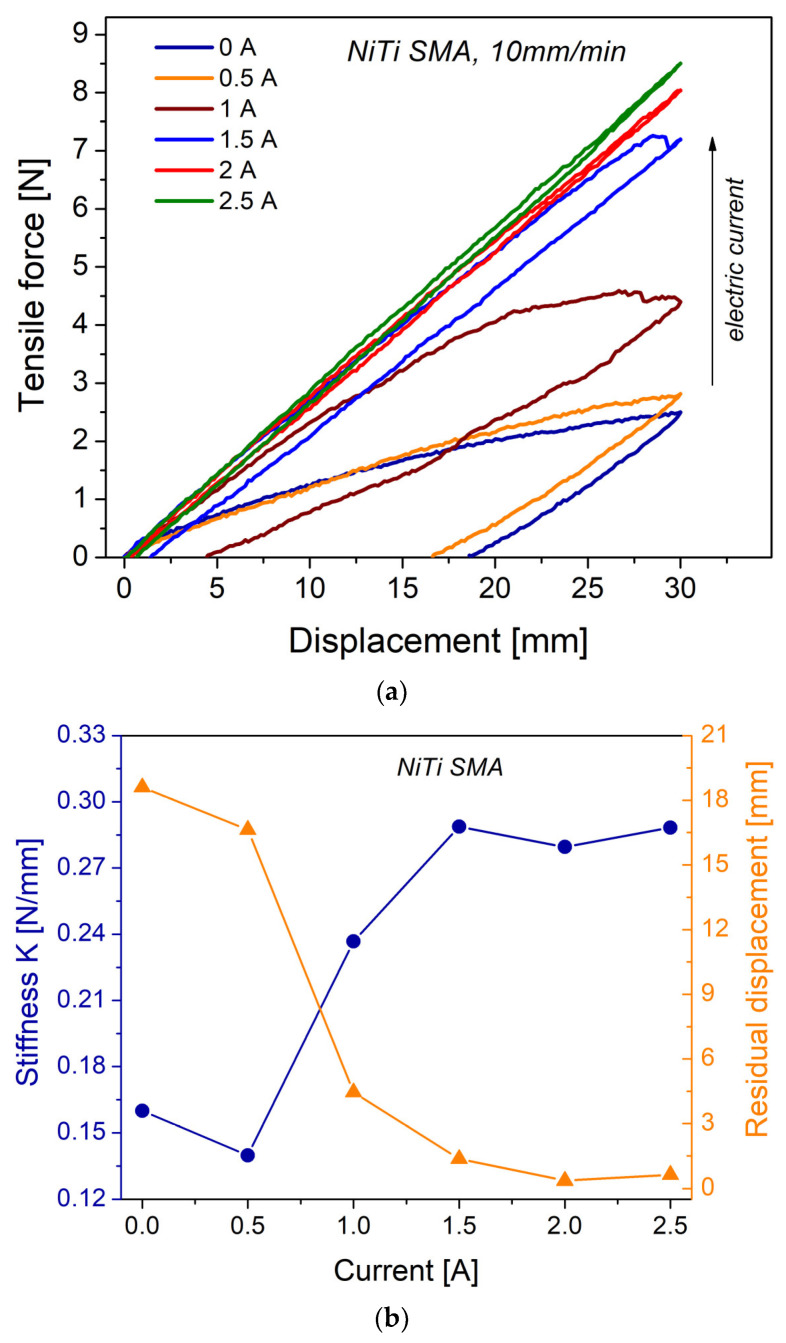
(**a**) Loading–unloading tests of the NiTi SMA spring at different current levels; (**b**) stiffness and residual displacement calculated from the loading–unloading tests.

Figure 5a shows the mechanical response of loading–unloading cycles of the NiTi SMA springs. The steel spring does not exhibit hysteresis; therefore, the results are not presented here. A notable hysteresis for NiTi SMA is observed, which translates into a high damping capacity, compared to conventional metals and alloys. For the level of displacement to which the springs were subjected, as the electric current increases, the area enclosed in the loops decreases. This means that the NiTi SMA spring presents a variable damping (energy dissipation), which depends on the fraction of martensitic transformation obtained. A residual displacement occurs after the spring has been loaded, which can be recovered or returned to its original shape by simple heating; see Figure 5b. The shape memory effect is obtained by passing from a low-temperature state (martensite phase) to a high-temperature state (austenite phase), by adding thermal energy to the coil spring or by applying an electric current that generates heat by the Joule effect [16]. It should be emphasized that the results obtained are a function of both the crystalline structure of the NiTi SMA and the geometry of the spring in question.

For a displacement of 30 mm, as the temperature increases, the force needed to develop actuation also increases (i.e., 2.5 N at 0 A, 8.5 N at 2.5 A). This also impacts the stiffness of the coil spring, which is shown in Figure 5b. In this sense, for the NiTi SMA, a change in stiffness means a change in the crystalline structure. According to the DSC results and temperature measurement in the spring, 0 A corresponds to the martensite phase and 2.5 A to the austenite phase. From stiffness K, it is possible to calculate the shear modulus G, using the equation K = dG/8NC^3^, where d is the diameter coil wire, N is the active number of coils, and C is the spring index. The estimated value for the martensitic state is G_M_ = 13.40 GPa and that for the austenitic state is G_A_ = 24.12 GPa; these values are close to those reported in the literature [18].

### 3.2. Dynamic Mechanical Analysis of NiTi SMA

**Figure 6:**
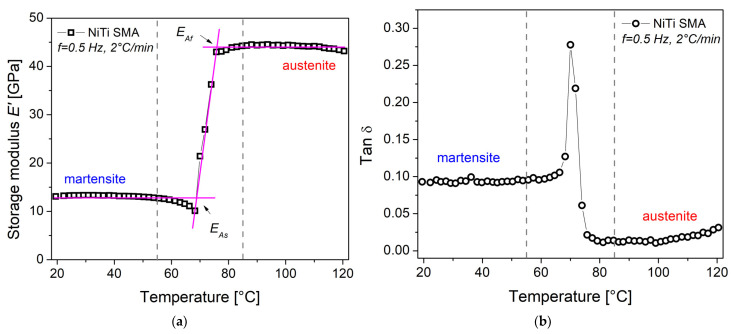
DMA results as function of temperature: (**a**) storage modulus E^′^; (**b**) tan δ.

Due to its sensitivity to atomic mobility, DMA is an experimental technique that allows characterizing the dissipation or partial energy storage in materials, mainly polymers and metals, when a periodic mechanical stimulus is applied. Atomic mobility could be related to the rate at which a portion of energy has been lost or stored in the material [24,27]. Figure 6a shows the real part of the complex elastic modulus, E^′^, as a function of temperature, under isochronous conditions. An increase in storage modulus E^′^ is observed with increasing temperature, from an almost constant value of 13.3 GPa to 44.3 GPa. This increase, 3.33 times, is due to a reverse martensitic transformation, which consists of a structural rearrangement of the low-temperature phase, martensite, to the high-temperature phase, austenite. These observations are in accordance with the results in Figure 5b. Around 70 °C, a slight decrease in the modulus occurs, which is presumed to be due to the movement of dislocations or interfaces, stress relaxation, or a combination thereof [30]. From these results, the values for transformation temperatures were calculated: austenite start E_As_ = 69 °C and austenite finish E_Af_ = 75.7 °C.

Figure 6b shows tan δ = E^″^/E^′^ as a function of temperature. The parameter tan δ is expressed as the ratio between the energy dissipated by the material and the energy stored, and from an engineering point of view, it is related to the damping capacity of mechanical vibrations. Three regions are qualitatively identified in the experimental results [27,31,32]. The first region, from 20 to 55 °C, corresponds to the damping produced by the movement of interfaces of the martensite variants. This magnitude is greater in comparison with the conventional metals and alloys [32]. In the second region, from 55 to 85 °C, the greatest energy dissipation occurs, due to the solid-state phase transformation from martensite to austenite. In the third region, above 85 °C, the material is in the austenite phase and therefore the dissipation of energy tends to a minimum.

### 3.3. Comparison between FZM and Experimental Data

**Figure 7:**
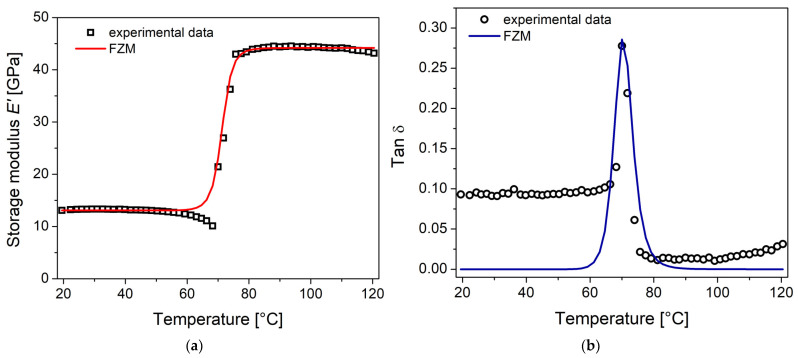
Comparison of the experimental data of DMA with predictions from the FZM: (**a**) storage modulus E^′^; (**b**) tan δ.

The Equations (3) and (4) obtained from the FZM are used for the modeling of the experimental data obtained by DMA for the NiTi SMA. Figure 7a shows a good agreement between the experimental data and theoretical predictions of the storage modulus E^′^. The model involves cooperative movements of the internal damping phenomenon in the NiTi SMA. Table 2 presents the parameters calculated for the description of the mechanical relaxation of the NiTi SMA. The activation energy parameters are consistent with the reported values [27,32]. The fractional order α=0.29 is associated with the martensite phase and the fractional order β=0.67 is associated with the austenite phase. The fractional order is related to the atomic mobility of the mechanical relaxation (martensitic phase transformation). When the fractional order approaches 1, the energy dissipation increases (viscous behavior) and, on the contrary, when this parameter tends to 0, the stored energy (elastic behavior) prevails over the dissipated one. Therefore, it is stated that β>α and that these parameters can be used to represent the damping process that occurs in the SMAs due to the martensite–austenite phase transformation in a solid state. Concerning tan δ, shown in Figure 7b, the model does not adequately follow the peak. This could be explained because other relaxation phenomena, at low and high temperatures, are not considered in the FZM. According to San Juan and Nó [31], the internal friction, due to the martensitic transformation in NiTi alloys, is composed of three contributions: transient, phase transition, and intrinsic damping processes.

**Figure 8:**
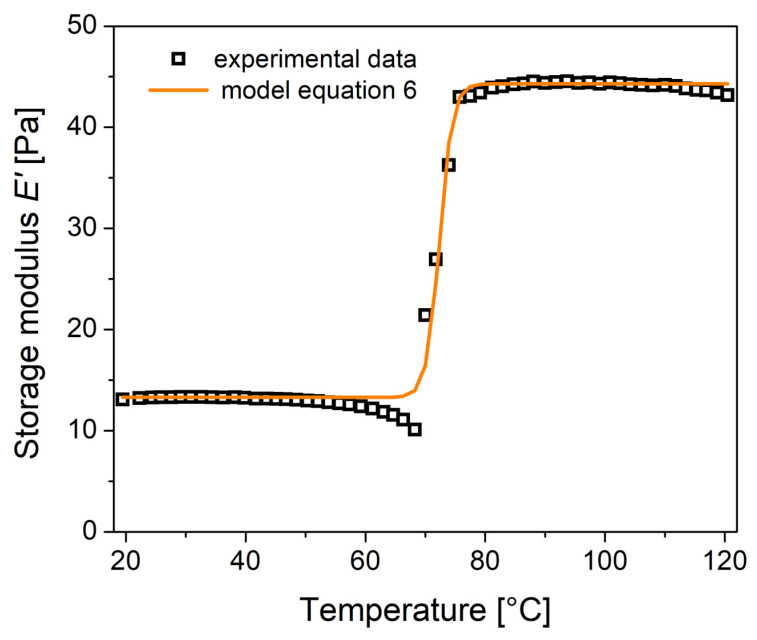
Comparison of the experimental data of DMA with prediction from Equation (6).

In order to compare the results obtained by the FZM, the following model, based on the work of Ikuta et al. for SMAs [33,34], is proposed for the phenomenological description of temperature-dependent storage modulus E^′^.

(6) $${E^{\prime }=E}_{A}+\frac{E_{M}-E_{A}}{{1+\exp[K}_{m}\left(T-T_{m}\right)]}$$

where E_M_ is the modulus of the SMA in the martensite phase, E_A_ is the modulus in the austenite phase, K_m_ = 6.2/(E_Af_ − E_As_) is a coefficient related to the growth rate, and T_m_ = (E_As_ + E_Af_)/2 is the mean transformation temperature of the SMA in the heating process. By considering that E_M_ = 13.3 GPa, E_A_ = 44.3 GPa, K_m_ = 0.925/°C, and T_m_ = 72.35 °C, in Figure 8 the comparison with the prediction of Equation (6) is presented; a good description of experimental data is obtained. The evaluation of Equations (3) and (6) for E^′^ results is in good agreement, so we calculate the root mean squared error (RMSE), which denotes the goodness-of-fit. The value for Equation (3) results in RMSE = 1.41 and that for Equation (6) results in RMSE = 1.09. This means that for NiTi SMA, the model of Equation (6) better represents the experimental data, since it has a smaller number of parameters. The advantage of the Fractional Zener Model (FZM) is the prediction of the viscoelastic behavior for the NiTi SMA over a wide spectrum of frequencies and temperatures. The proposed model, Equation (6), can be used as a simple way to work only with the real component of the complex elastic modulus, as a function of the temperature. However, this model neglects the damping process of the martensitic transformation in the NiTi SMA.

### 3.4. Vibratory Response of Mass-Spring NiTi System

**Figure 9:**
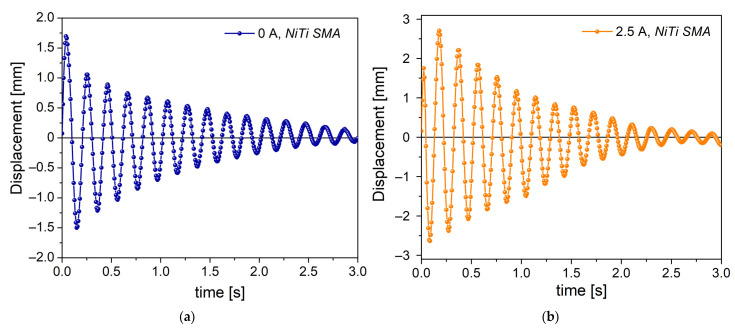
Free vibration response of mass-spring NiTi system: (**a**) state A at 0 A; (**b**) state B at 2.5 A.

Figure 9a shows the free vibration response of the mass-spring NiTi system at 0 A. An underdamped oscillatory response of the vibration is observed, that is, the vibration decays exponentially over time. In vibratory systems, the damping capacity can come from different sources, for example, the interaction with the surrounding medium or from the internal friction. In this condition, the NiTi SMA spring is in the martensite phase, a crystalline structure with damping resulting from the stress-induced movement of the martensite–martensite interfaces (twin movement) [1,5,15]. Posteriorly, the spring is heated by applying an electrical current of 2.5 A. The current generates the Joule effect, which increases the temperature of the coil spring, allowing it to contract and pass to the austenite phase. After this, the mass-spring NiTi system begins to vibrate freely. Figure 9b shows the free vibration result, where an underdamped response is observed, similar to that in Figure 9a. From the classical point of view, these vibratory responses can be interpreted under the framework of second-order differential equations; however, the nonlinearities associated with the material under study generate certain discrepancies [35,36]. Future work will be carried out to model the vibration response using the fractional calculus approach.

From the data in Figure 9a,b, the vibrational parameters were calculated: natural frequency f_n_ and stiffness K (see Table 3). It was determined that the stiffness increased by 14.12% when passing from state A to state B. This is consistent with the data of Figure 5b and Figure 6a, where the stiffness and modulus increase as the temperature of the NiTi SMA increases. As the mass is constant in the vibratory system, dynamic stiffness produces a change in the natural frequency, generating an increase of 6.83%. A similar finding was obtained by Adeodato et al. [37].

In engineering applications, working with materials outside the linear region allows, in terms of their properties, to extend the range of operation. However, it makes its use difficult and complicates the process of mechanical design. In the case of SMAs, it makes it possible to have materials with variable mechanical properties (stiffness and damping) within a certain range, which from the control point of view opens the possibility of designing adaptive mechanical systems, for instance, vibration absorbers in mechanical devices and structures [15,16]. This, in turn, implies difficulties for its use, since these characteristics must be generated and maintained in appropriate ranges, for example: (a) maintaining an adequate temperature range so as not to deprogram the material or deform it outside the desired range; (b) heat and cool in a controlled way to obtain a predictable mechanical response; (c) heat or cool with the required rate to be able to pass from one state to another (martensite–austenite or vice versa) and not slow down the mechanical processes.

## 4. Conclusions

The shape memory effect in the NiTi SMA coil springs was corroborated at room temperature. The results obtained by DMA show that this effect is independent of the geometry. The mechanical response of the NiTi SMA springs gives rise to a variable stiffness, which can be adjusted by different stimuli, such as an electric current or temperature. A ratio of 3.33 was obtained for the storage modulus at austenite/martensite phases, and the damping capacity presented a maximum energy dissipation at 70 °C. The complex elastic modulus was interpreted under the fractional calculus framework, where a fractional order of 0.29 was obtained at low temperatures, while a value of 0.67 was obtained at high temperatures. These parameters are associated with the atomic mobility of the martensite–austenite phase transformation. A simple model is proposed for the phenomenological interpretation of the martensitic transformation. The NiTi SMA spring reported in this work can be used for designing mechanical systems with potential applications in shock and vibration control.

## Figures and Tables

**Table 1:** Chemical composition of NiTi SMA.

Element	Weight (%)	Atomic (%)
Ni	45.05	50.12
Ti	54.95	49.88

**Table 2:** Parameters obtained from the FZM.

Parameters	Values	Units
E_U_	1.31 × 10^10^	Pa
E_0_	4.42 × 10^10^	Pa
A	0.29	-
Β	0.67	-
E_aa_	0.66	eV
E_ab_	0.57	eV
τ_0a_	1 × 10^−13^	s
τ_0b_	1 × 10^−13^	s
T*	99	°C
T_0_	28	°C

**Table 3:** Vibrational parameters computed from the vibration data.

	Natural Frequency (Hz)	Stiffness (N/mm)
State A	4.98	0.256
State B	5.32	0.292

## Data Availability

Data is available within the manuscript.
